# Development of XMRV producing B Cell lines from lymphomas from patients with Chronic Fatigue Syndrome

**DOI:** 10.1186/1742-4690-8-S1-A230

**Published:** 2011-06-06

**Authors:** Francis Ruscetti, Vincent C Lombardi, Michael Snyderman, Dan Bertolette, Kathryn S Jones, Judy A Mikovits

**Affiliations:** 1 Center for Cancer Research NCI-Frederick, Frederick, Maryland, 21702, USA; 2 Whittemore Peterson Institute, University of Nevada, Reno, Nevada, 89557, USA; 3 Department of Medicine, State University of New York at Buffalo, Buffalo, New York, USA; 4 Basic Research Program, SAIC-Frederick, Inc., NCI-Frederick, Frederick, Maryland, 21702, USA

## 

Previous studies have shown that CFS patients have an increased incidence of lympho-proliferative malignancy compared to the normal population [1]. While the incidence rate of non-Hodgkin’s lymphoma in the United States is 0.02%, nearly 5% of the CFS patients developed the disease. Additionally, development of cancer coincides with an outgrowth of gamma delta T cells with specific clonal T-cell receptor gamma rearrangements. We hypothesized that infection with XMRV and/or other viruses can trigger a dysregulated immune response which favors the development of B-cell lymphoma.

In a study of 300 CFS patients, 13 developed lymphoproliferative disorders. Of those tested, 11/11 were positive for XMRV and 9/9 positive for clonal TCR gamma rearrangements. Spontaneous development of four B cells lines occurred during culture of cells from CFS patients. Three developed from B cells isolated from the peripheral blood (two of whom had B cell lymphoma) and one from a bone marrow biopsy. For all four lines, the B cells have a mature CD20+, CD23+ phenotype and produce infectious XMRV at a titer of >10^6^ infectious units/ml. Virus production occurred despite extensive hypermutation of the proviruses in these cells by APOBEC3G.. Therefore XMRV infection may accelerate the development of B cell malignancies by either indirect chronic stimulation of the immune system and/or by direct infection of the B-cell lineage . Since viral load in peripheral blood is low, these data suggest that B cells in tissues such as spleen and lymph nodes could be an in vivo reservoir for XMRV.
